# Transfer of beef bacterial communities onto food-contact surfaces

**DOI:** 10.3389/fmicb.2024.1450682

**Published:** 2024-10-07

**Authors:** Giselle K. P. Guron, Jennifer M. Cassidy, Chin-Yi Chen, George C. Paoli

**Affiliations:** ^1^Oak Ridge Institute for Science and Education, U.S. Department of Agriculture, Agricultural Research Service, Eastern Regional Research Center, Wyndmoor, PA, United States; ^2^Molecular Characterization of Foodborne Pathogens Research Unit, U.S. Department of Agriculture, Agricultural Research Service, Wyndmoor, PA, United States; ^3^Characterization and Interventions for Foodborne Pathogens Research Unit, U.S. Department of Agriculture, Agricultural Research Service, Eastern Regional Research Center, Wyndmoor, PA, United States

**Keywords:** beef, meat tissue, biofilm, attachment, food-contact surface, *Pseudomonas*

## Abstract

**Introduction:**

Food spoilage and pathogenic bacteria on food-contact surfaces, especially biofilm-forming strains, can transfer to meats during processing. The objectives of this study were to survey the bacterial communities of beef cuts that transfer onto two commonly used food-contact surfaces, stainless steel (SS) and high-density polyethylene (HDPE) and identify potentially biofilm-forming strains.

**Methods:**

Top round, flank, chuck, and ground beef were purchased from 3 retail stores. SS and HDPE coupons (approximately 2cm × 5cm) were placed on beef portions (3h, 10°C), after which, the coupons were submerged halfway in PBS (24h, 10°C). Bacteria from the beef cuts and coupon surfaces (*n* = 3) were collected, plated on tryptic soy agar plates and incubated (5 days, 25°C). Bacterial isolates were identified by 16S rRNA gene amplicon sequencing and assayed for biofilm formation using a crystal violet binding (CV) assay (72h, 10°C). Additionally, beef and coupon samples were collected for bacterial community analysis by 16S rRNA gene amplicon sequencing.

**Results and discussion:**

Sixty-one of 972 beef isolates, 29 of 204 HDPE isolates, and 30 of 211 SS isolates were strong biofilm-formers (Absorbance>1.000 at 590 nm in the CV assay). Strong-binding isolates identified were of the genera *Pseudomonas*, *Acinetobacter*, *Psychrobacter*, *Carnobacterium*, and *Brochothrix*. Coupon bacterial communities among stores and cuts were distinct (*p* < 0.001, PERMANOVA), but there was no distinction between the communities found on HDPE or SS coupons (*p* > 0.050, PERMANOVA). The bacterial communities identified on the coupons may help determine the communities capable of transferring and colonizing onto surfaces, which can subsequently cross-contaminate foods.

## Introduction

Microbial transfer to/from and colonization of food-contact surfaces is a concern in the meat industry because the resident microbiota of facilities influences the quality and safety of final products (Hultman et al., 2015; Stellato et al., 2016). Operational taxonomic units (OTUs) or amplicon sequence variants (ASVs) are often shared among several kinds of beef cuts and surfaces used throughout beef production, distribution, and storage (De Filippis et al., 2013; Hultman et al., 2015; Stellato et al., 2016; Zwirzitz et al., 2020), including spoilage organisms such as *Pseudomonas*, *Brochothrix*, and lactic acid bacteria (Russo et al., 2006; Nychas et al., 2008; Ercolini et al., 2009; Casaburi et al., 2011; Chaillou et al., 2015). While cleaning and disinfecting can shift the microbiota of surfaces (Maillet et al., 2021), spoilage organisms can persist on production surfaces (Marouani-Gadri et al., 2009; Fagerlund et al., 2017; Wang et al., 2018; Maes et al., 2019; Bjorge Thomassen et al., 2023) and may continue to influence the microbiomes of meat products through cross contamination.

An important factor contributing to the persistence of bacterial populations on food processing surfaces is the formation of biofilms, which are typically comprised of a multi-species community of organisms within extracellular polymeric substances (Costerton et al., 1995). Biofilm formation allows these communities to survive different chemical and physical antimicrobial procedures often performed by the industry (Wang, 2019). However, prior to biofilm formation on food-contact surfaces, bacteria must transfer from one source and then attach onto the new surface. When contact between raw beef is made, not only can bacterial cells deposit onto the food contact surface, but residual proteins and lipids from the meat itself also transfer and condition the surface (Dourou et al., 2011; Kimkes and Heinemann, 2020). The transfer process can be affected by many environmental factors, such as hydrophobicity and topography of the recipient surface (Van Houdt and Michiels, 2010; Berne et al., 2018).

Details of which organisms from food can transfer onto surfaces upon contact are poorly understood, though there have been studies on how pathogen-inoculated beef transfer their inoculum onto surfaces (Flores et al., 2006; Dourou et al., 2011; Beauchamp et al., 2012). Understanding how different species transfer onto surfaces is important since multiple biofilm-forming species have been isolated from the meat processing equipment (Møretrø et al., 2013; Roder et al., 2015). Additionally, mixed-species biofilms formed by isolates from beef-contact surface can accumulate greater biomass than single species biofilms (Roder et al., 2015; Lapointe et al., 2019; Sadiq et al., 2023). Since biofilm communities comprise a mixture of strains that may affect beef quality or safety directly (Roder et al., 2015; Lapointe et al., 2019), it is important to identify biofilm-forming aerobic bacteria to determine what species are more likely to contribute to biofilms on food-contact surfaces.

The first objective of this study was to use culture-dependent and -independent methods to characterize the bacterial communities of 4 cuts of beef from 3 retail establishments, and then determine how the different community structures affect bacterial transfer onto 2 different food contact surface materials at a temperature commonly used in the meat industry. The two materials used were stainless steel (SS), which was smooth to the touch and eye, and high-density polyethylene (HDPE), which was visibly rough. The second objective was to determine which of the isolates from the beef or the surfaces were biofilm-forming organisms, and whether the transferring procedure helps select for those organisms.

## Materials and methods

### Beef preparation

Beef cuts were purchased from 3 retail stores (stores A, B, and C), stored at 4°C, and sampled within 24 h. A description of the packaging for each product is provided in Supplementary Table S1. Each beef cut from each retail store was sampled in triplicate. Three separate portions of beef (approximately 25 g for each of the triplicate samples) were aseptically cut from the packaged beef and transferred to sterile 400-ml blender bags with filters (Fisher Scientific, Hampton, NH). Buffered peptone water (BPW, Neogen, Lansing, MI) was added to the bag [beef/BPW, 1/10 (w/v)] and the samples were blended with a Stomacher® 400 (Seward, Islandia, NY) for 30 s at 230 rpm. Serial dilutions were prepared using BupH™ phosphate-buffered saline (PBS, Thermo Scientific™, Waltham, MA) and 100 μl of appropriate dilutions was spread onto Bacto trypic soy agar (TSA, Becton Dickinson, Franklin Lakes, NJ) plates for microbial isolation and enumeration. Plates were incubated at 25°C for 5 days.

Fourteen milliliter of the blended sample was collected for 16S rRNA gene amplicon sequencing. Large particulate and insoluble fat were removed by centrifuging for 2 min at 1,500 × *g* in an Avanti J-25 centrifuge (Beckman Coulter, Indianapolis, IN) to reduce particulates that can interfere with the DNA extraction protocol. Ten milliliter of supernatant was transferred to a new tube (avoiding the visible muscle tissue and fat), and the cells were collected by centrifugation for 5 min at 10,000 × *g* in an Avanti J-25 centrifuge (Beckman Coulter). After discarding the supernatant, the cell pellet was frozen at −80°C until DNA extraction.

### Coupon preparation

Stainless steel (SS) and high-density polyethylene (HDPE) coupons (5.1 × 2.1 cm and 5.2 × 2.6 cm, respectively) were soaked in 1% (v/v) Liquinox (Alconox, White Plains, NY) for 1 h and rinsed with distilled water. Each side was then sprayed with 100% ethanol and air-dried prior to autoclaving wrapped in an aluminum foil pack (Beauchamp et al., 2012). After coupons were autoclaved and cooled to room temperature, the foil package containing the coupons was opened inside a biosafety cabinet (Purifier Logic+, LabConco, Kansas City, MO) and the coupons were exposed to UV light for 15 min. The coupons were carefully flipped using metal tweezers that had also been autoclaved and exposed to UV light to induce DNA damage, and the 15-min UV light exposure was repeated for the other side of the coupons.

Three of each coupon type were aseptically placed onto the beef so that one side was in contact with the product at 10°C for 3 h. The coupons contacted one beef cut from all stores on 1 day. Triplicate negative control SS and HDPE coupons that were cleaned and autoclaved, but made no contact with beef were included on each of the 4 days. The coupons were then transferred into 50-ml conical tubes and stored vertically so that half of the coupon was submerged in PBS to facilitate bacterial attachment at the air-liquid interface (Sauer et al., 2022): 15 ml for SS coupons and 35 ml for HDPE coupons. The tubes containing the coupons were incubated statically at 10°C for 24 h.

The coupons were then each transferred to new 50-ml conical tubes containing 20 ml of 37°C sterilized, ultrapure type I (18 mega ohm) water (Purelab Flex 2, Elga, United Kingdom) and were shaken vigorously by hand for 10 s to remove loose meat and fat particulates. The rinsed coupons were each transferred to a new 50-ml conical tube containing 40 ml BPW and 10 3-mm sterile borosilicate glass beads (Sigma Aldrich, St. Louis, MO), and shaken on a vortex mixer (Vortex-Genie 2, Scientific Industries, Inc., Bohemia, NY) as described by Beauchamp et al. (2012) at maximum speed for 2 min. One-hundred microliters of the BPW containing the dislodged cells was then spread onto tryptic soy agar. An additional 1 ml of the BPW, in triplicate, was centrifuged in 1.5-mL Eppendorf tubes for 3 min at 13,000 × *g* to concentrate the cells 10-fold at the bottom of the tube. Nine hundred microliter of the supernatant was discarded and the remaining 100 μl was plated. The plates were incubated aerobically at 25°C for 5 days. Additionally, 14 ml of the BPW containing the dislodged cells was transferred to a 15-ml conical tube and centrifuged for 5 min at 10,000 × *g* in an Avanti J-25 centrifuge (Beckman Coulter). The supernatant was discarded, and the cell pellet was stored at −80°C until DNA extraction.

### DNA extraction and 16S rRNA gene amplicon sequencing

The PowerFood® Microbial DNA Isolation Kit (Qiagen, Germantown, MD) was used to isolate DNA from all samples, with the additional step of warming the samples at 70°C for 10 min prior to the bead beating step. The DNA extracts were shipped to Clear Labs (Menlo Park, CA) for 16S rRNA gene amplicon sequencing. Libraries were prepared with the 300-cycle MiSeq Reagent Kit v3 (Illumina, Inc., San Diego, CA), and 2 × 250 bp paired-end sequencing was performed using lllumina MiSeq instrument (Illumina, Inc., San Diego, CA).

The trimmed, demultiplexed 16S rRNA gene sequences were received from Clear Labs, and QIIME2-Amplicon-2024.5 (Bolyen et al., 2019) was used for processing. The Silva SSU N99 database 138.1 was downloaded in July 2024 (Quast et al., 2013), and the rescript plugin was used for parsing, culling low-quality and short sequences, and dereplicating using the least common ancestor method (Robeson et al., 2021). The DADA2 plugin (Callahan et al., 2016) was used to merge 180-bp reads and remove chimeric sequences using the default settings. Features were then classified as ASVs (Pedregosa et al., 2011; Bokulich et al., 2018). Contaminant reads were identified (decontam-identify) and removed (decontam-remove) based on a decontam score threshold of 0.1 using the quality-control plugin. Chloroplast, mitochondria, and singleton sequences were then removed.

### Collection and identification of bacterial isolates

After 5 days of incubation on TSA, isolated colonies from beef samples and coupons from all 3 stores were individually inoculated into 5 ml tryptic soy broth (TSB), grown at 25°C until visibly turbid, and frozen in 15% glycerol at −80°C. A total of 1,387 isolates were collected and frozen back.

In addition, 533 isolates collected from beef and the SS and HDPE coupons from Store B were identified by Sanger sequencing the amplicon of the16S rRNA gene. Three milliliter of TSB was inoculated with a single colony of a beef or coupon isolate and incubated at 10°C with shaking (180 rpm) for 3 days. The cells from 1 ml of culture were collected by centrifugation and extracted using 50 μl of PrepMan Ultra according to the manufacturer’s instructions (Applied Biosystems, Foster City, CA).

For the DNA samples prepared from SS and HDPE coupon isolates, the 16S rRNA gene was first amplified using primers EubA (5′-AAGGAGGTGATCCANCCRCA) and EubB (5′-AGAGTTTGATCMTGGCTCAG; Cottrell and Kirchman, 2000) yielding an approximate 1,500-bp product. PCR was performed using GoTaq® Green Master Mix (Promega, Madison, WI) in 25 μl with final primer concentrations at 1 μM each. The amplification conditions with the ProFlex PCR System (Applied Biosystems™, Waltham, MA) were as follows: 95°C for 90 s, then 30 cycles of 95°C for 30 s, 55°C for 45 s, and 72°C for 60 s, and a final extension at 72°C for 5 min. Agencourt® AMPure® XP paramagnetic particles (Beckman Coulter, Indianapolis, IN) were used for PCR clean up. DNA sequencing of the PCR product was performed using primers EubA, EubB, 519F (5′-GWATTACCGCGGCKGCTG), and 519R (5′-CAGCMGCCGCGGTAATWC) for each DNA sample (Irwin et al., 2008).

For bacteria isolated directly from beef, the 16S rRNA gene was first amplified using primers 338F (5′-ACTCCTACGGGAGGCAGCAG) and 1046R (5′-CGACAGCCATGCASCACCT; Youssef et al., 2009) yielding an approximately 725 bp PCR product (16S rRNA gene regions V3-V6). PCR was performed using GoTaq® Green Master Mix (Promega, Madison, WI) in 25 μl with final primer concentrations at 1 μM each. The amplification conditions were as follows: 95°C for 90 s, then 30 cycles of 95°C for 30 s, 60°C for 45 s, and 72°C for 60 s, and a final extension at 72°C for 5 min. Agencourt® AMPure® XP was used for PCR clean up. This primer pair was validated using select coupon isolates to assure the same genera were identified as the sequences amplified by EubA and EubB. DNA sequencing of PCR products was performed using primers 338F and 1046R.

The nucleotide sequencing of all PCR amplicons was performed in-house using the BigDye™ Terminator v3.1 Cycle Sequencing Kit (Applied Biosystems™, Waltham, MA) in 20 μl reactions containing the following mixture: 1.0 μl cleaned PCR product, 1.0 μl BigDye™ Terminator, 1.0 μl 3.2-μM primer, 3.5 μl 5× BigDye™ Terminator v3.1 Sequencing Buffer, and 13.5 μl water. The following temperature parameters were used for cycle sequencing: 1 cycle at 95°C for 5 min, followed by 30 cycles at 96°C for 10 s, 55°C for 5 s, and 60°C for 4 min. Agencourt® CleanSEQ (Beckman Coulter, Indianapolis, IN) was used for BigDye™ clean-up prior to loading onto a SeqStudio Genetic Analyzer (Applied Biosystems™, Waltham, MA) for nucleotide sequence determination. The final sequences were trimmed and assembled using Sequencher® 5.4.6 (Gene Codes Corporation, Ann Arbor, MI). The 16S rRNA gene sequences were searched using BLAST against the National Center for Biotechnology Information 16S ribosomal RNA sequences database (Altschul et al., 1990) using Geneious Prime 2023.0.1. Bowtie2 was also used with Geneious Prime to align Sanger sequences to Illumina sequences (Langmead and Salzberg, 2012).

### Screening of biofilm formation by bacterial isolates

Isolates from all 3 stores were screened for biofilm formation. Prior to assessing biofilm formation via crystal violet binding assays, bacterial cultures were precultured in tryptic soy broth (TSB) at 10°C at 180 rpm for 3 days. Then 1 μl from each culture was used to inoculate 100 μl of no salt TSB (NSTSB; 17.0 g Bacto™ Tryptone)/L (Becton Dickinson, Franklin Lakes, NJ) and 3.0 g Bacto™ Soytone /L (Becton Dickinson, Franklin Lakes, NJ) and incubated at 10°C in 96-well flat-bottom polystyrene plates (TPP Tissue Culture Test Plates; Techno Plastic Products, Trasadingen, Switzerland) for 3 days. Absorbance was measured at 600 nm using a Safire^2^ microplate reader (Tecan, Männedorf, Switzerland) to determine bacterial growth. An ELx405 Automatic Plate Washer (BioTek, Winooski, VT) was used to remove the culture from the 96-well plate and rinse the plates 3 times with 300 μl ultrapure type I water to remove loosely adhered cells. After rinsing, the remaining attached cells were stained with 200 μl of 0.1% crystal violet (w/v; Sigma Aldrich, St. Louis, MO) for 30 min, rinsed 3 more times with water, and destained with 95% ethanol for 30 min. Absorbance was measured at 590 nm using the Safire^2^.

### Statistical analysis

Wilcoxon tests, Kruskal-Wallis tests and Spearman’s correlation coefficients were performed using R 3.5.1 and above (R Core Team, 2018). Figures were generated using Microsoft Office and ggplot2 (Wickham et al., 2019). Multiple comparisons were adjusted for *p*-value using the Bonferroni correction. For enumeration, values below the LOD (<0.57 log CFU/cm^2^ for SS and <0.47 log CFU/cm^2^ for HDPE) were inputted as 0 values. Diversity analyses were performed using QIIME2-2024.5 (Bolyen et al., 2019). R packages qiime2R (Bisanz, 2018), phyloseq (McMurdie and Holmes, 2013), vegan (Oksanen et al., 2024), and VennDiagram (Chen, 2022) were used for further analysis and data visualization. Significance was defined at *p* < 0.050.

## Results

### Enumeration of bacteria from beef cuts and coupons

**Figure 1:**
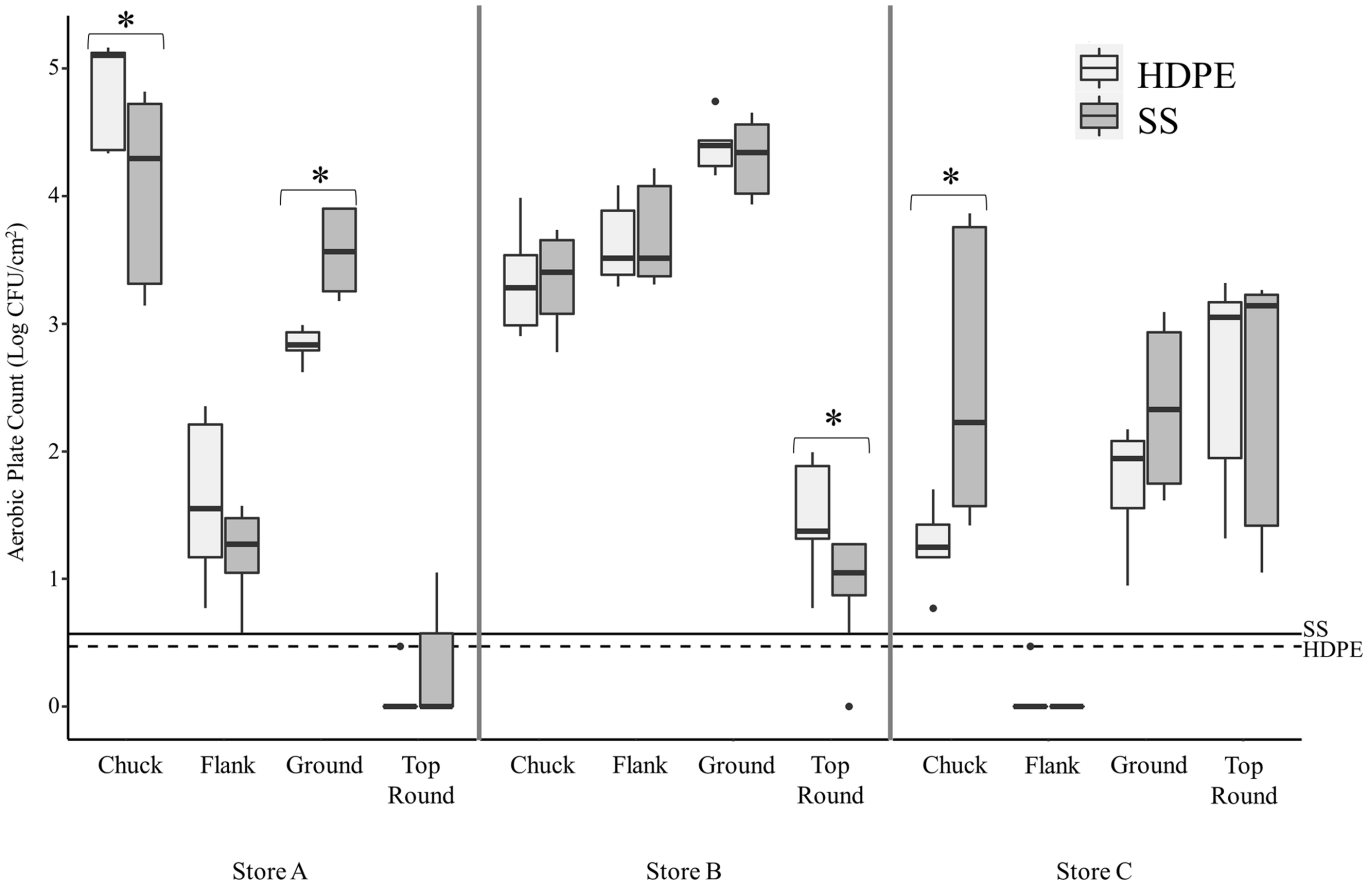
Transfer of beef-associated bacteria to stainless steel (SS) and high-density polyethylene (HDPE) coupons (*n* = 3). The aerobic plate counts (APC, Log CFU/cm^2^) on TSA are presented from coupons that contacted the specified beef cuts from three retail supermarkets. The maximum, minimum, third quartile, first quartile, median, and outliers are displayed. The asterisks (*) indicate where there was a significantly difference in APC between the SS and HDPE coupons that contacted the same beef sample (*p* < 0.05, Wilcoxon). The limit of detection for experiments using the SS and HDPE coupons are shown using solid and dashed horizontal lines, respectively.

The average aerobic plate count (APC) from beef cuts ranged from 3.27 ± 0.44 log CFU/g (from store C flank steak) to 7.70 ± 0.20 log CFU/g (from store A chuck steak; Table 1). All the beef appeared and smelled fresh and acceptable for consumption, despite the large differences in APC. Bacterial cells were enumerable for most coupons, but some coupon contained bacteria below the detection limits (LOD; <0.47 log CFU/cm^2^ for HDPE; <0.57 log CFU/cm^2^ for SS). The average APC from HDPE coupons that were above the LOD ranged from 1.30 ± 0.28 log CFU/cm^2^ (Store C chuck) to 4.85 ± 0.38 log CFU/cm^2^ (Store A chuck; Figure 1). For Store A top round and Store C flank steak, only 1 CFU was detected from 1 of 3 HDPE coupons. The APC from other HDPE coupons that contacted other beef cuts were above LOD. The APC detected from SS coupons were in range with those from the HDPE, between averages of 1.21 ± 0.34 log CFU/cm^2^ (from Store A flank) to 4.31 ± 0.28 log CFU/cm^2^ (Store B ground beef). Colonies were detected from only 1 of 3 SS coupons from Store A top round (0.73 CFU/cm^2^) and no colonies were isolated from any SS coupon from Store C flank steak. No colonies were detected on any control coupons that did not contact beef. There is a strong positive correlation between the APC from the beef itself and the APC from HDPE coupons (*p* < 0.001, *ρ* = 0.81, Spearman’s) and the APC from SS coupons (*p* < 0.001, *ρ* = 0.93, Spearman’s), indicating that a greater bacterial load present on the initial food results in a greater transfer of bacterial cells onto both surface materials.

**Table 1:** Mean aerobic plate counts on TSA at 25°C from beef cuts purchased from 3 different retail stores (*n* = 3).

Retail store	Aerobic plate counts from cuts of beef (Mean log CFU/g ± SD)			
	Chuck	Flank	Ground	Top Round
Store A	7.70 ± 0.20	4.89 ± 0.13	6.53 ± 0.10	4.07 ± 0.20
Store B	6.46 ± 0.41	6.19 ± 0.32	7.61 ± 0.11	5.82 ± 0.23
Store C	6.37 ± 0.33	3.27 ± 0.44	5.32 ± 0.11	6.00 ± 0.14

There were some significant differences found between the 2 coupon types within each beef cut, but only one difference was greater than 1 log in magnitude (Figure 1). The mean of the APC from SS was greater than from HDPE from Store C chuck by 1.2 log CFU/cm^2^ (*p* < 0.003, Wilcoxon).

### Identification of bacterial isolates from Store B

Store B products were further studied because all beef cuts contained APC above LOD. There were 533 isolates from Store B beef and coupons that were randomly selected for identification using Sanger sequencing of 16S rRNA gene amplicons. Eighteen genera were identified from 533 colonies picked from all store B samples (Table 2; Dataset S1). *Pseudomonas* and *Carnobacterium* were detected directly on all beef cuts, and *Pseudomonas* was also detected on both coupon types that contacted all beef cuts. The most frequently identified related species were *P. deceptionensis* (82 out of 245 *Pseudomonas* isolates) and *P. weihenstephanensis* (70 out of 245 isolates).

**Table 2:** Ratios of strong binders* over the total number of colonies identified from Store B beef or coupons.

Genus	Biofilm formation by bacterial genera isolated												Total
	(# Strong biofilm-forming isolates^a^/# Total number of isolates tested)												
	Chuck			Flank			Ground			Top Round			
	Direct	HDPE^b^	SS^c^	Direct	HDPE	SS	Direct	HDPE	SS	Direct	HDPE	SS	
*Plantibacter*	0	0	0	0	0	0	0	0	0	0/3	0	0	0/3
*Galactobacter*	0	0	0	0	0	0	0	0	0	0/1	0	0	0/1
*Flavobacterium*	0	0	0	0	0	0	0	0	0	0/10	0	0	0/10
*Pedobacter*	0	0	0	0	0	0	0	0	0	0/1	0	0	0/1
*Sphingobacterium*	0	0	0	0	0	0	0	0	0	0/3	0	0	0/3
*Brochothrix*	0/4	0/1	0/1	0/4	0	0	0	0	0	0/4	0/3	2/6	2/23
*Staphylococcus*	0/1	0	0	0	0	0	0	0	0	0	0	0	0/1
*Carnobacterium*	0/19	0/1	0/2	0/8	1/5	0/5	0/12	0	0/1	0/8	0/1	1/2	2/64
*Latilactobacillus*	0/1	0	0	0/2	0	0	0/3	0/1	0	0	0/5	0	0/12
*Leuconostoc*	0/1	0	0	0	0	0	0	0	0	0/2	0/3	0	0/6
*Lactococcus*	0/4	0/2	0/1	0/1	0	0/1	0/4	0	0	0/15	0/2	0/1	0/31
*Brucella*	0	0	0	0	0	0	0	0	0	0/1	0	0	0/1
*Herbaspirillum*	0	0	0	0	0	0	0	0	0	0/1	0	0	0/1
*Janthinobacterium*	0	0	0	0	0	0	0	0	0	0/1	0	0	0/1
*Yersinia*	0	0	0	0	0	0	0/9	0	0	0	0	0	0/9
*Acinetobacter*	0/1	0	0	1/6	0	0	3/9	0/5	0	0	0	0	4/21
*Psychrobacter*	1/7	0	0	1/1	0	0	0	0	0	0	0	0	2/8
*Pseudomonas*	7/32	3/12	2/12	7/49	2/6	2/8	12/54	5/12	2/5	6/38	3/6	6/11	57/245
Unknown	0/5	2/5	1/1	3/19	0/13	0/10	0/2	1/3	5/15	0/6	3/6	2/7	17/92
Total	8/75	5/21	3/17	12/90	3/24	2/24	15/93	6/21	7/21	6/94	6/26	11/27	84/533

The ratios of strong binders* over the total number of isolates from beef or coupons tested at 10°C are presented for the bacterial genera identified.

a Strong binders had an absorbance > 1.00 at 590 nm in the crystal violet binding assays.

b HDPE, high-density polyethylene coupons.

c SS, stainless steel coupon.

### Potential biofilm-forming isolates from beef and coupons

Crystal violet assays were performed with all 1,387 isolates from all stores to measure bacterial attachment to 96-well polystyrene plate surfaces. Isolates yielding an absorbance >1.0 at 590 nm were categorized as strong binders and potential biofilm formers (Table 3). Overall, 61 of 972 beef isolates (6.3%), 29 of 204 HDPE isolates (14.2%), and 30 of 211 SS isolates (14.2%) were strong binders. Store B had the highest proportions of strong binders, with 11.6% (41/352) of all beef isolates, 21.7% (20/92) HDPE isolates, and 25.8% (23/89) SS isolates. Within Store B (Table 2), the highest proportion of strong binders originated from SS coupons that contacted the top round (40.7%; 11/27), whereas the lowest proportion of strong binders originated from the top round itself (6 of 94 isolates; 6.4%). *Pseudomonas* was the most common strong binder, with 23.3% (57/245) of these isolates being strong binders. The strong binding strains from Store B top round SS coupons were *Pseudomonas*, *Brochothrix*, and *Carnobacterium*. No other *Brochothrix* strains were strong binders, and only one other *Carnobacterium* strain in Store B HDPE from flank was a strong binder. Strong binding *Brochothrix* and *Carnobacterium* originated from coupons, but not directly from the beef. Conversely, the strong binding *Acinetobacter* and *Psychrobacter* only originated from beef, but not from coupons. Genera were detected on coupons if they were also detected directly from beef with one exception. *Latilactobacillus* (formerly *Lactobacillus*) was detected on HDPE, but not on the top round on which that coupon made contact. The beef cut and coupons yielding the greatest number of genera was the top round.

**Table 3:** Biofilm formation by bacterial isolates from beef or coupon surfaces.

Store	Beef cut	Biofilm formation by bacteria isolated from various cuts of beef (#strong biofilm-forming isolates^a^/#total number of isolates tested)			
		Directly from beef	From HDPE^b^	From SS^c^	Total
A	Chuck	9/96	3/20	5/17	17/133
	Flank	1/96	0/24	0/24	1/144
	Ground	2/96	0/20	0/20	2/136
	Top Round	6/96	0/3	0/9	6/108
B	Chuck	8/75	5/21	3/17	16/113
	Flank	12/90	3/24	2/24	17/138
	Ground	15/93	6/21	7/21	28/135
	Top Round	6/94	6/26	11/27	23/147
C	Chuck	0/42	0/11	0/10	0/63
	Flank	1/16	0/1	0/0	1/17
	Ground	1/96	3/21	0/18	4/135
	Top Round	0/82	3/12	2/24	5/118
	Total	61/972	29/204	30/211	120/1387

The proportions of strong binders^a^ over the total number of isolates from beef or coupons tested at 10°C are presented for each type of beef cut from each retail store.

a Strong binders had an absorbance > 1.00 at 590 nm in the crystal violet binding assays.

b HDPE, high-density polyethylene coupons.

c SS, stainless steel coupon.

### Bacterial compositions and diversity of the microbiota from beef cuts and coupons

In addition to identifying isolates from Store B, the bacterial communities from each beef cut and coupon sample were determined by culture-independent 16S rRNA gene amplicon sequencing. A total of 3,316,090 high-quality sequencing reads were generated, 867,893 of which were directly from beef, 1,625,886 were from the coupons that contacted the beef, and 822,311 were from negative control samples. The beef samples averaged 24,110 ± 21,369 reads, while the coupon samples averaged 22,583 ± 17,149 reads. A total of 748 and 2,742 ASVs were identified among beef and coupon samples, respectively. Additionally, there were 1,213 ASVs identified from control coupons (that had no contact with beef) and 315 ASVs from sterile BPW and the blank DNA extracts prepared without the addition of reagents besides those included in the kit. There were many taxa from the negative control coupons shared with the coupons that contacted beef (Supplementary Figure S1). Since the negative control coupons overlapped with many of the beef coupon samples (Supplementary Figure S2), the coupon dataset was decontaminated by identifying and removing the contaminant ASVs from the control coupons. The beef and decontaminated coupon samples were rarefied to 1,260 and 2,320 reads, respectively, for comparison.

**Figure 2:**
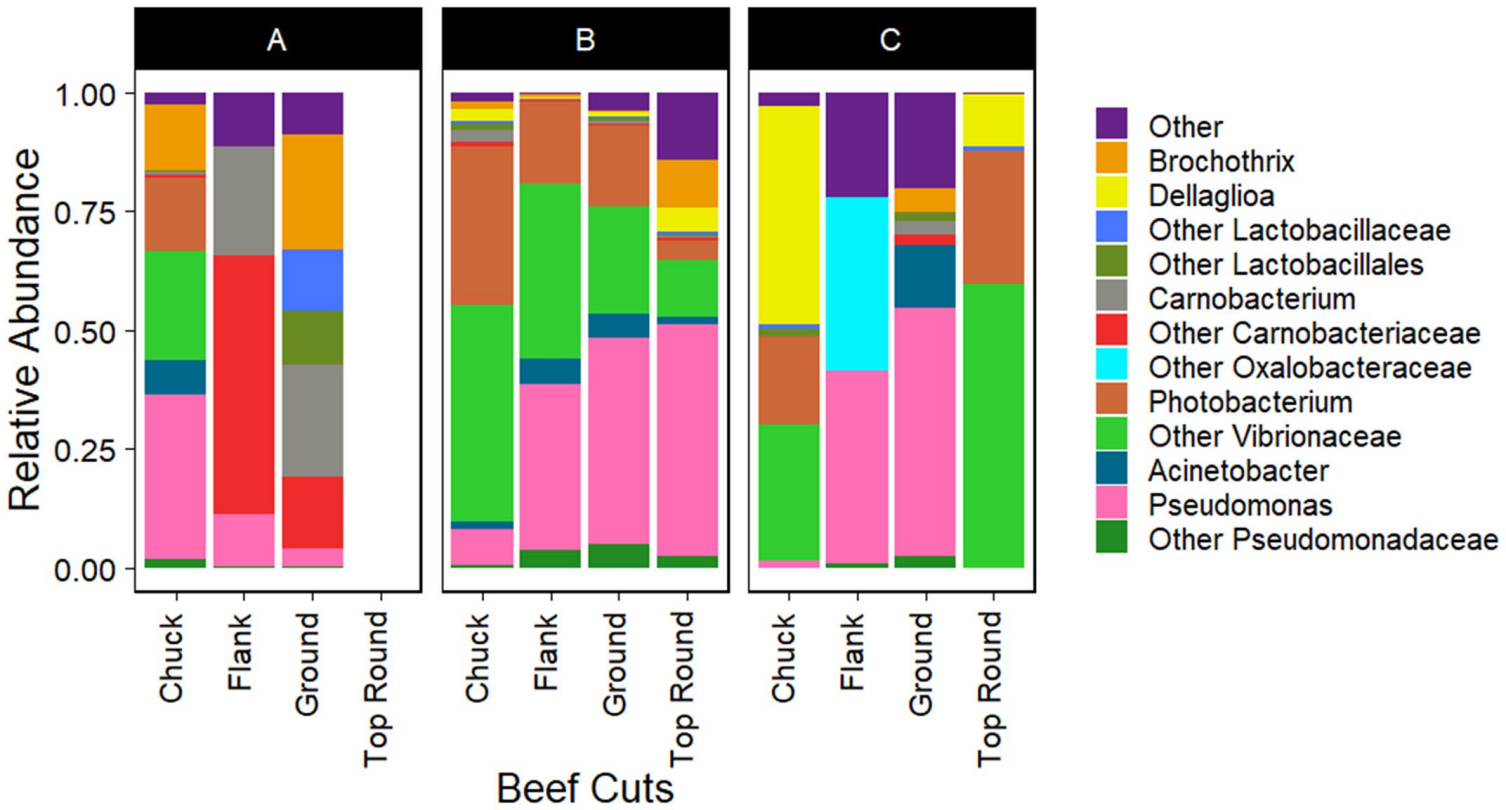
Taxonomic composition (rarefied to 1,260 reads) of beef microbiota as aligned with Silva 138.1. All experiments were done in triplicate except Store A flank and Store C flank where *n* = 2 and Store A top round where *n* = 0 due to rarefaction.

**Figure 3:**
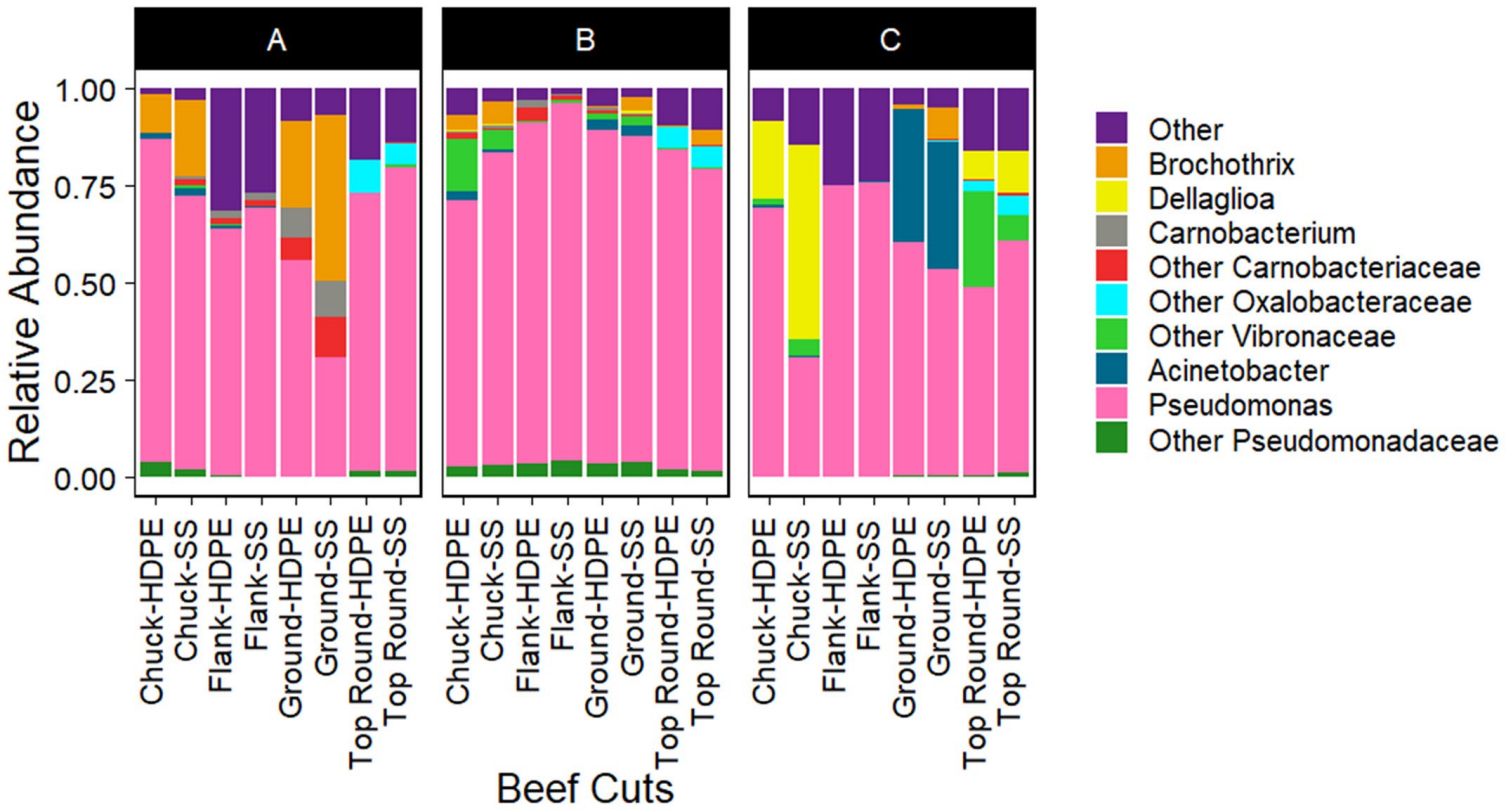
Taxonomic composition (rarefied to 2,320 reads) of coupon microbiota after contact with beef cuts. All experiments were done in triplicate except HDPE from Store B top round and HDPE and SS from Store C top round where *n* = 1 due to rarefaction.

*Gammaproteobacteria* and *Bacilli* were the dominant classes in the bacterial populations from both the beef and the microbiota that transferred onto coupons (Figures 2, 3). *Photobacterium*, other *Vibrionaceae*, *Pseudomonas*, other *Pseudomonadaceae*, *Dellaglioa* (formerly *Lactobacillus*), *Carnobacterium*, and other *Carnobacteriaceae* were detected in over 70% of the beef samples, and *Acinetobacter* and *Pseudomonas* were detected in over 73% of coupon samples.

Interestingly, the *Actinomycetia* isolates, *Plantibacter*, *Galactobacter*, the *Alphaproteobacteria* isolate, *Brucella*, and the *Betaproteobacteria* isolates, *Herbaspirillum*, and *Janthinobacterium*, were identified directly from the top round using culture-dependent methods (Table 2), but they were not identified via Illumina sequencing of the top round bacterial communities. Bowtie2 was used to double check if any reads from the top round beef samples aligned with these Sanger sequences, but none completely aligned.

### Diversity of beef bacterial communities

**Figure 4:**
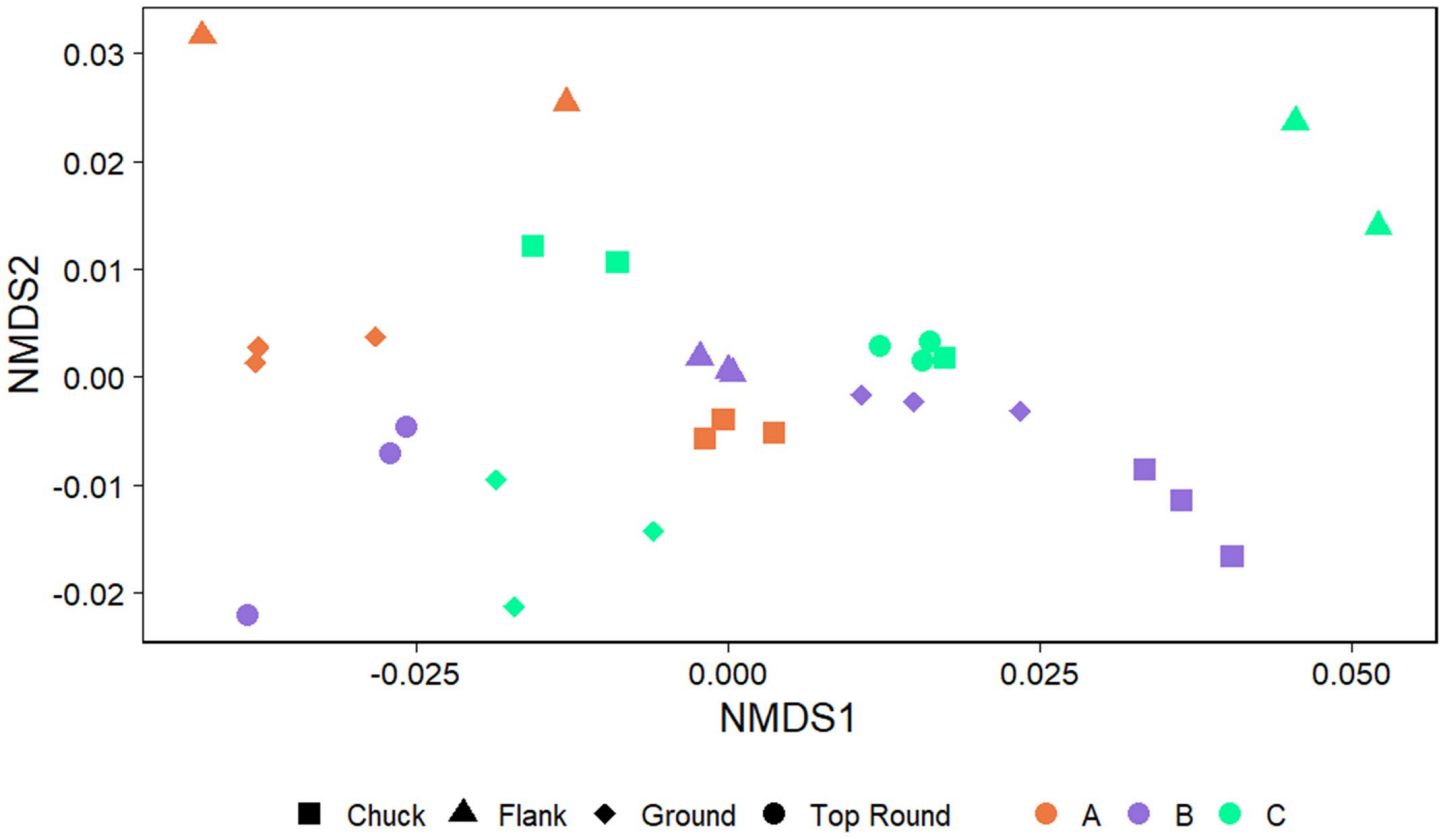
Nonmetric multidimensional scaling (NMDS) plots generated from weighted Unifrac distance matrices of microbiota directly from beef cuts from retail stores. Stores were differentiated by color while cuts were differentiated by shape. All experiments were done in triplicate except Store A flank and Store C flank where *n* = 2 and Store A top round where *n* = 0 due to rarefaction.

The communities of beef samples were significantly separated by store (*p* = 0.017, PERMANOVA), but not by beef cut (*p* > 0.050; Figure 4). There were significant differences in ASV richness and evenness among the microbiota identified directly from the beef cuts (*p* < 0.001, ANOVA; Supplementary Table S2). Flank from Store A and B had the lowest richness compared to chuck, ground, and top round. Flank also generally shared the fewest ASVs among the rest of the beef cuts within each of the stores (Supplementary Figure S3). Store B, where all the retail meats are displayed the glass, shared the highest ASVs compared to the two stores that individually wrapped their meat products. Ground beef also shared the most ASVs with chuck and top round in Stores A and B.

**Figure 5:**
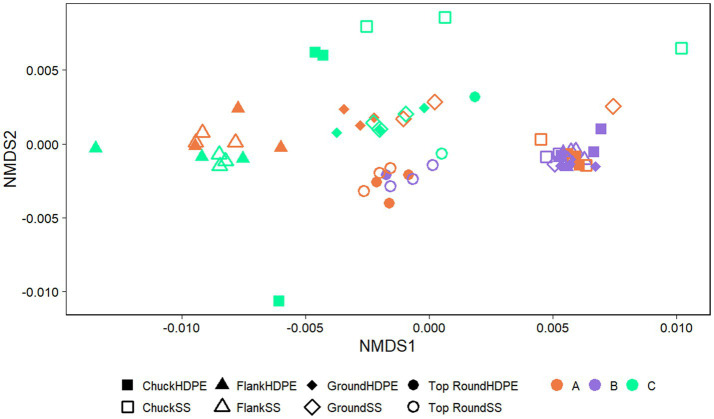
NMDS plots generated from weighted Unifrac distance matrices of microbiota on HDPE or SS coupons transferred from beef cuts from retail stores. Stores were differentiated by color while cuts were differentiated by shape. All experiments were done in triplicate except HDPE from Store B top round and HDPE and SS from Store C top round where *n* = 1 due to rarefaction.

The coupon communities were significantly separated by store (*p* < 0.001) and beef cut (*p* < 0.001; Figure 5), but no significant separation was observed between the coupon type, HDPE or SS (p > 0.050). There were significant differences in richness and evenness on the coupons (Supplementary Table S3; *p* < 0.050, ANOVA). Most notably, the microbiota identified on coupons that contacted Store A chuck was the lowest in richness than those from flank and top round. Store B chuck, flank, and ground generally also had the lowest Chao1 compared to the other stores.

## Discussion

Bacterial communities from beef were transferred onto surfaces at 10°C, a temperature that simulates a meat processing facility. There were some differences in the bacteria enumerated from SS and HDPE coupons as determined by APCs on TSA, but there were no significant differences in alpha or beta diversity between the coupon types from culture-independent 16S rRNA gene analysis. There was no consistent difference in enumeration of bacteria between the two coupon surfaces, and there was no clear distinction between the SS and HDPE communities, so it is possible some of the differences between bacterial counts for a certain coupon and beef cut combinations are bacterial strain specific as was observed with the transfer of *Salmonella* between beef and these 2 surfaces (Gkana, 2017). Ultimately, culturing was necessary to differentiate bacteria between the coupons that contacted the beef and the microbial DNA detected on control coupons.

Identifying cultured isolates informed the validity of the ASVs identified through culture-independent 16S rRNA gene analysis. For example, while culture-independent sequencing identified several *Lactobacillales* genera from both SS and HDPE coupon samples that contacted beef and the control coupons, the culturing methods provided assurance that these *Lactobacillales* were present on the coupons that contacted the store B beef cuts. *Pseudomonas* was isolated from all store B samples, which was similarly reflected through 16S rRNA gene amplicon sequencing. In contrast, when there was little or no transfer of bacteria from beef onto the coupons, contaminant DNA from the extraction kit, autoclaved media, and equipment most likely amplified during the sequencing process (Salter et al., 2014). Longer autoclave times or UV exposure times may have been necessary to reduce contaminant DNA further on the coupons and other consumable lab materials (Gefrides et al., 2010). In a study that characterized the microbiota attached to SS or tri-polyurethane coupons from a salmon processing facility the authors examined communities from negative controls from the DNA extraction process, excluding those OTUs from subsequent analyses (Maillet et al., 2021). The authors did not specifically identify OTUs from contaminant DNA on cleaned coupons. The present study is the first to demonstrate that the cleaned coupons could have residual bacterial DNA even if no viable organisms were detected. Interestingly, both the present study and the work of Maillet et al. (2021) from the salmon facility had greater reads produced from the exposed coupons than the animal tissue, despite there being greater APC from the animal tissue over the coupon communities. A recent metagenomics study identified a significantly greater alpha diversity from non-food-contact surfaces over food-contact surfaces and cheese products, though no culturing was performed to assess the viable bacteria on any surfaces (De Filippis et al., 2024).

Moreover, while culture-independent methods identify taxa not easily culturable, there were examples in the present study where the culturing method identified taxa not detected by high throughput sequencing. For the majority of Store B genera identified from the beef and coupon combinations, there were more isolates of each genus from beef than there were from the coupons, which was to be expected given the differential sample sizes. However, the one case where *Latilactobacillus* was only isolated from the HDPE rather than the top round is exceptional considering that this genus was not even detected on the coupons by high throughput sequencing above 1% in relative abundance. The optimal media for *Lactobacillaceae* is de Man, Rogosa and Sharpe broth, and its growth is substantially lower in TSA (Calix-Lara et al., 2012), so the growth of multiple isolates on TSA was especially interesting. This further underscores the need for more studies comparing the microbiota characterized by culturing to culture-independent methods.

Conversely, *Photobacterium* and other *Vibrionaceae* were identified through 16S rRNA gene sequencing of the DNA directly from the microbiota from all store B samples, but both taxa were not identified through culture-dependent methods. Many species within the *Vibrionaceae* require lower temperatures, higher salinity, and antibiotics to outcompete other foodborne organisms (Donovan and van Netten, 1995; Hilgarth et al., 2018). The greatest ASV richness in the microbiota in store B beef cuts was from top round, and this trend was also observed in the isolates selected by culturing. Both culture-dependent and -independent methods yielded results that one or the other missed, which is consistent with past studies that compared identifying cultured isolates with molecular methods applied to DNA extracted directly from beef such as cloning of 16S rRNA genes (Olofsson et al., 2007) and PCR-denaturing gradient gel electrophoresis (Pennacchia et al., 2011).

Bacteria transferred from retail beef products onto SS and HDPE coupons was dependent on both store and cut, but not on the surface material. Similarly, it has been reported that the APC and quantity of inoculated *E. coli* O157:H7 that transferred onto either SS or HDPE from ground beef was not significantly different between the two surface materials (Dourou et al., 2011). However, Dourou et al. (2011) observed that the different media used impacted the quantities of bacteria transferred onto the surfaces. In contrast, a study with bologna inoculated with *Listeria monocytogenes* found there was a greater transfer rate between the bologna and SS compared to HDPE (Rodriguez and McLandsborough, 2007). However, the latter study was performed under a much shorter contact time of 30 s and at a temperature not below 15°C, unlike the present study and the ground beef study (Dourou et al., 2011) which allowed hours of contact time at the lower temperatures. Even though SS and HDPE generally differ in hydrophobicity (Shi and Zhu, 2009), the proteins and lipids that condition the coupons may have more impact on transfer of microbiota onto the surface with longer contact time at colder temperatures.

More *Pseudomonas* isolates that had transferred onto the coupons were also identified as strong binders compared to other genera identified. *Pseudomonadales* are consistently detected on multiple meat processing surfaces that have been surveyed at different locations (Marouani-Gadri et al., 2009; Hultman et al., 2015; Stellato et al., 2016; Fagerlund et al., 2017; Maes et al., 2019; Wagner et al., 2020; Zwirzitz et al., 2020), most likely because many strains can attach and form biofilms on these surfaces under cooler conditions (Morimatsu et al., 2012; Liu et al., 2015; Zhu et al., 2019). *Brochothrix* and *Carnobacterium* are other potential psychrophilic spoilage organisms (Russo et al., 2006; Nychas et al., 2008; Ercolini et al., 2009; Casaburi et al., 2011; Maes et al., 2019) that are attached to the coupon surfaces. *Brochothrix* was even present among 80% of the biofilms sampled in a meat processing facility, highlighting the importance of this genera (Wagner et al., 2020). *Acinetobacter* and *Psychrobacter* are also organisms often found on spoiled meats (Gennari et al., 1992; De Filippis et al., 2013; Chaillou et al., 2015), as well as on conveyer belts from meat processing plants (Fagerlund et al., 2017; Wang et al., 2018). The 5 strong-binding genera from beef cuts in the present study were also found in surveys of the surfaces of meat processing facilities (Stellato et al., 2016; Maes et al., 2019).

*Pseudomonadales* and certain *Firmicutes* (also known as *Bacillota*), such as *Carnobacterium* and *Brochothrix,* should be targeted for cleaning and sanitizing strategies to prevent their attachment after contact with beef. Psychrotrophic *Pseudomonas* are especially vital to control since many species are associated with spoilage in refrigerated meat products (Wickramasinghe et al., 2019) and are often found on meat processing facilities all over the world (De Filippis et al., 2013; Hultman et al., 2015; Stellato et al., 2016; Zwirzitz et al., 2020). While these genera are important to note, considering that these genera were also detected from the other stores that had fewer strong binders, specific practices of different meat processing facilities should be explored to further understand how more biofilm-formers appeared to be selected for in one store over the other two. Future studies comparing *Pseudomonas* strains from these different stores could provide insight to this discrepancy. Further studies are also necessary to determine how potential foodborne spoilage bacteria or bacterial pathogens are transferred between beef and beef processing surfaces and how these organisms interact with the existing surface microbiota.

## Data Availability

The datasets presented in this study can be found in online repositories. The names of the repository/repositories and accession number(s) can be found in the article/Supplementary material. Illumina sequences can be downloaded from https://www.ncbi.nlm.nih.gov/bioproject/PRJNA658004/.

## Author’s note

Mention of trade names or commercial products in this publication is solely for the purpose of providing specific information and does not imply recommendation or endorsement by the US Department of Agriculture. USDA is an equal opportunity provider and employer.
