# Supplementary material for: Transfer of beef bacterial communities onto food-contact surfaces
Source: Front Microbiol. 2024 Oct 7;15:1450682. doi: 10.3389/fmicb.2024.1450682 (PMC11491791; doi:10.3389/fmicb.2024.1450682)
Supplement: Supplementary file 1 [file Data_Sheet_1.zip › Table 1 - 2024-09-11T100908.716.DOCX]

Supplementary Material

Table S1. Description of packaging for all the beef cuts purchased from the 3 retail stores.

|  | Store |  | Description of Retail Beef Packaging for Cuts of Beef | | | | | | |  |
| --- | --- | --- | --- | --- | --- | --- | --- | --- | --- | --- |
|  |  |  | Chuck |  | Flank |  | 85% Lean Ground |  | Top Round |  |
|  | Store A*^a^* |  | Plastic wrapped, Styrofoam bottom with absorbent pad; sliced into steaks |  | Plastic wrapped, Styrofoam bottom with absorbent pad; one slab |  | Plastic wrapped, Styrofoam bottom with absorbent pad |  | Plastic wrapped, Styrofoam bottom with absorbent pad, thinly sliced |  |
|  | Store B*^b^* |  | Wrapped in butcher’s paper with plastic sheet on top; one roast |  | Wrapped in butcher’s paper with plastic sheets rolled around one slab |  | Placed in plastic bag wrapped in butcher’s paper |  | Wrapped in butcher’s paper with plastic sheets between thin slices |  |
|  | Store C*^a^* |  | Plastic wrapped, Styrofoam bottom with absorbent pad; sliced into steaks |  | Vacuum packed with bottom absorbent pad; one slab |  | Plastic wrapped, Styrofoam bottom with absorbent pad |  | Plastic wrapped, Styrofoam bottom with absorbent pad; one slab |  |

*^a^*Purchased pre-packaged from meat cooler, sampled within a few days of purchase prior to “use by” date.

*^b^*Purchased from meat counter and packaged by butcher
