# Supplementary material for: Transfer of beef bacterial communities onto food-contact surfaces
Source: Front Microbiol. 2024 Oct 7;15:1450682. doi: 10.3389/fmicb.2024.1450682 (PMC11491791; doi:10.3389/fmicb.2024.1450682)
Supplement: Supplementary file 1 [file Data_Sheet_1.zip › Table 2 - 2024-09-11T100909.589.DOCX]

Supplementary Material

Table S2. Αlpha-diversity of microbiota directly from beef cuts, calculated from 16S rRNA gene amplicon sequencing (99% identity OTU cutoff).

|  |  |  | Mean±SD |  |  |  | |  | |
| --- | --- | --- | --- | --- | --- | --- | --- | --- | --- |
| Store | Cut |  | Chao1 |  | Shannon Index |  | | Pielou's Evenness | |
| A | Chuck (n=3) |  | 57.61±4.07^cd^ |  | 4.27±0.06^ab^ |  | | 0.74±0.01^ab^ | |
|  | Flank (n=2)*^*^* |  | 11.50±4.95^g^ |  | 2.54±0.92^e^ |  | | 0.72±0.13^abc^ | |
|  | Ground (n=3) |  | 51.06±2.87^cde^ |  | 4.40±0.13^ab^ |  | | 0.79±0.02^a^ | |
| B | Chuck (n=3) |  | 75.37±6.28^b^ |  | 3.63±0.22^bcd^ |  | | 0.60±0.03^cd^ | |
|  | Flank (n=3) |  | 20.33±1.15^fg^ |  | 3.08±0.09^de^ |  | | 0.71±0.03^abc^ | |
|  | Ground (n=3) |  | 63.70±5.50^bc^ |  | 4.09±0.10^abc^ |  | | 0.70±0.01^abc^ | |
|  | Top Round (n=3) | | 41.50±5.07^de^ |  | 4.08±0.35^abc^ |  | | 0.77±0.06^ab^ | |
| C | Chuck (n=3) |  | 36.23±6.31^ef^ |  | 3.27±0.39^cde^ |  | | 0.64±0.06^bcd^ | |
|  | Flank (n=2)*^*^* |  | 100.50±11.31^a^ | | 4.76±0.05^a^ | |  | | 0.73±0.01^abc^ |
|  | Ground (n=3) |  | 42.79±4.24^de^ |  | 4.24±0.11^ab^ |  | | 0.79±0.02^c^ | |
|  | Top Round (n=3) | | 22.07±9.60^fg^ |  | 2.46±0.30^e^ |  | | 0.57±0.03^d^ | |
| Controls | BPW (n=3) |  | 92.10±27.87 |  | 3.10±0.33 |  | | 0.54±0.03 | |
|  | Blank (n=3) |  | 68.25±36.42 |  | 2.85±0.13 |  | | 0.56±0.02 | |

*^*^*Samples were omitted due to rarefaction (1260 sequences)

^a-g^Different letters indicate significant differences within each alpha diversity metric among the beef cuts and store (p<0.050, ANOVA)
