# Supplementary material for: Transfer of beef bacterial communities onto food-contact surfaces
Source: Front Microbiol. 2024 Oct 7;15:1450682. doi: 10.3389/fmicb.2024.1450682 (PMC11491791; doi:10.3389/fmicb.2024.1450682)
Supplement: Supplementary file 1 [file Data_Sheet_1.zip › Table 3 - 2024-09-11T100910.589.DOCX]

Supplementary Material

Table S3 Alpha-diversity of microbiota transferred onto coupons from beef cuts, calculated from 16S rRNA gene amplicon sequencing.

|  |  |  |  | Mean±SD |  |  |  |  |
| --- | --- | --- | --- | --- | --- | --- | --- | --- |
| Store | Cut | Coupon |  | Chao1 |  | Shannon |  | Evenness |
| A | Chuck | HDPE (n=3) | | 25.67±8.50^c^ |  | 3.15±0.16^c^ |  | 0.69±0.04^a^ |
|  |  | SS (n=3) |  | 28.83±2.57^c^ |  | 3.31±0.28^c^ |  | 0.69±0.04^a^ |
|  | Flank | HDPE (n=3) | | 111.49±9.47^a^ |  | 4.15±0.25^a^ |  | 0.63±0.04^abc^ |
|  |  | SS (n=3) |  | 141.60±28.25^a^ | | 3.98±0.20^a^ |  | 0.59±0.01^abc^ |
|  | Ground | HDPE (n=3) | | 104.43±21.55^ab^ | | 4.07±0.30^ab^ |  | 0.65±0.03^ab^ |
|  |  | SS (n=3) |  | 77.63±47.33^ab^ |  | 3.88±0.42^ab^ |  | 0.66±0.06^ab^ |
|  | Top Round | HDPE (n=3) | | 77.56±31.86^bc^ | | 3.48±0.34^c^ |  | 0.57±0.01^c^ |
|  |  | SS (n=3) |  | 50.97±10.79^bc^ |  | 3.05±0.21^c^ |  | 0.55±0.01^c^ |
| B | Chuck | HDPE (n=3) | | 29.67±8.02^c^ |  | 3.39±0.18^c^ |  | 0.70±0.05^a^ |
|  |  | SS (n=3) |  | 29.67±6.11^c^ |  | 3.26±0.13^c^ |  | 0.67±0.05^a^ |
|  | Flank | HDPE (n=3) | | 27.00±1.00^c^ |  | 3.07±0.17^c^ |  | 0.65±0.05^abc^ |
|  |  | SS (n=3) |  | 22.00±1.00^c^ |  | 2.90±0.16^c^ |  | 0.65±0.03^abc^ |
|  | Ground | HDPE (n=3) | | 37.67±2.52^c^ |  | 3.12±0.21^c^ |  | 0.60±0.03^abc^ |
|  |  | SS (n=3) |  | 35.17±11.62^c^ |  | 3.18±0.18^c^ |  | 0.63±0.03^abc^ |
|  | Top Round | HDPE (n=1)^*^ | | 37.00^bc^ |  | 2.83^c^ |  | 0.54^bc^ |
|  |  | SS (n=3) |  | 59.48±3.46^bc^ |  | 3.36±0.19^c^ |  | 0.57±0.03^bc^ |
| C | Chuck | HDPE (n=3) | | 80.69±16.64^ab^ | | 2.97±0.89^c^ |  | 0.49±0.13^c^ |
|  |  | SS (n=3) |  | 88.03±62.15^ab^ | | 3.61±0.29^c^ |  | 0.63±0.08^c^ |
|  | Flank | HDPE (n=3) | | 105.37±28.45^ab^ | | 3.48±0.12^bc^ |  | 0.56±0.00^c^ |
|  |  | SS (n=3) |  | 83.83±22.44^ab^ |  | 3.38±0.02^bc^ |  | 0.56±0.02^c^ |
|  | Ground | HDPE (n=3) | | 90.51±12.99^ab^ |  | 4.05±0.15^a^ |  | 0.65±0.01^abc^ |
|  |  | SS (n=3) |  | 102.69±22.16^ab^ | | 4.16±0.20^a^ |  | 0.65±0.03^abc^ |
|  | Top Round | HDPE (n=1)^*^ | | 41.75^bc^ |  | 3.35^abc^ |  | 0.62^abc^ |
|  |  | SS (n=1)^*^ |  | 69.60^bc^ |  | 3.79^abc^ |  | 0.63^abc^ |

*Samples were omitted due to rarefaction (2320 sequences)

^a-c^Different letters indicate significant differences within each alpha diversity metric for beef cuts and store (p<0.050, ANOVA). There were no significant differences between coupon type (p>0.050).
