# Supplementary material for: Transfer of beef bacterial communities onto food-contact surfaces
Source: Front Microbiol. 2024 Oct 7;15:1450682. doi: 10.3389/fmicb.2024.1450682 (PMC11491791; doi:10.3389/fmicb.2024.1450682)
Supplement: Supplementary file 1 [file Data_Sheet_1.zip › Supplementary Figure 1.docx]

Supplementary Material


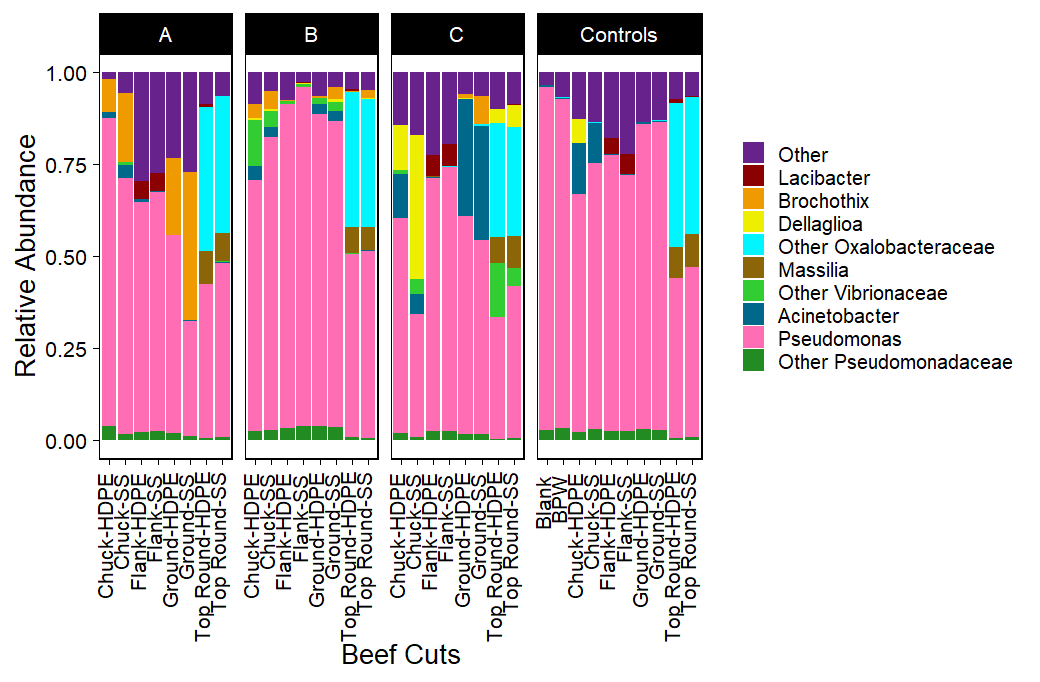


Figure S1. Taxonomic composition (rarefied to 2480 reads) of coupon microbiota after contact with beef cuts. Negative control coupons were processed on the same day as the experiment of each beef cut, but they did not make any contact with beef. All experiments were done in triplicate except HDPE from store B top round and HDPE and SS from store C top round where n=1 due to rarefaction.
