# Supplementary material for: Transfer of beef bacterial communities onto food-contact surfaces
Source: Front Microbiol. 2024 Oct 7;15:1450682. doi: 10.3389/fmicb.2024.1450682 (PMC11491791; doi:10.3389/fmicb.2024.1450682)
Supplement: Supplementary file 1 [file Data_Sheet_1.zip › Supplementary Figure 3.docx]

Supplementary Material


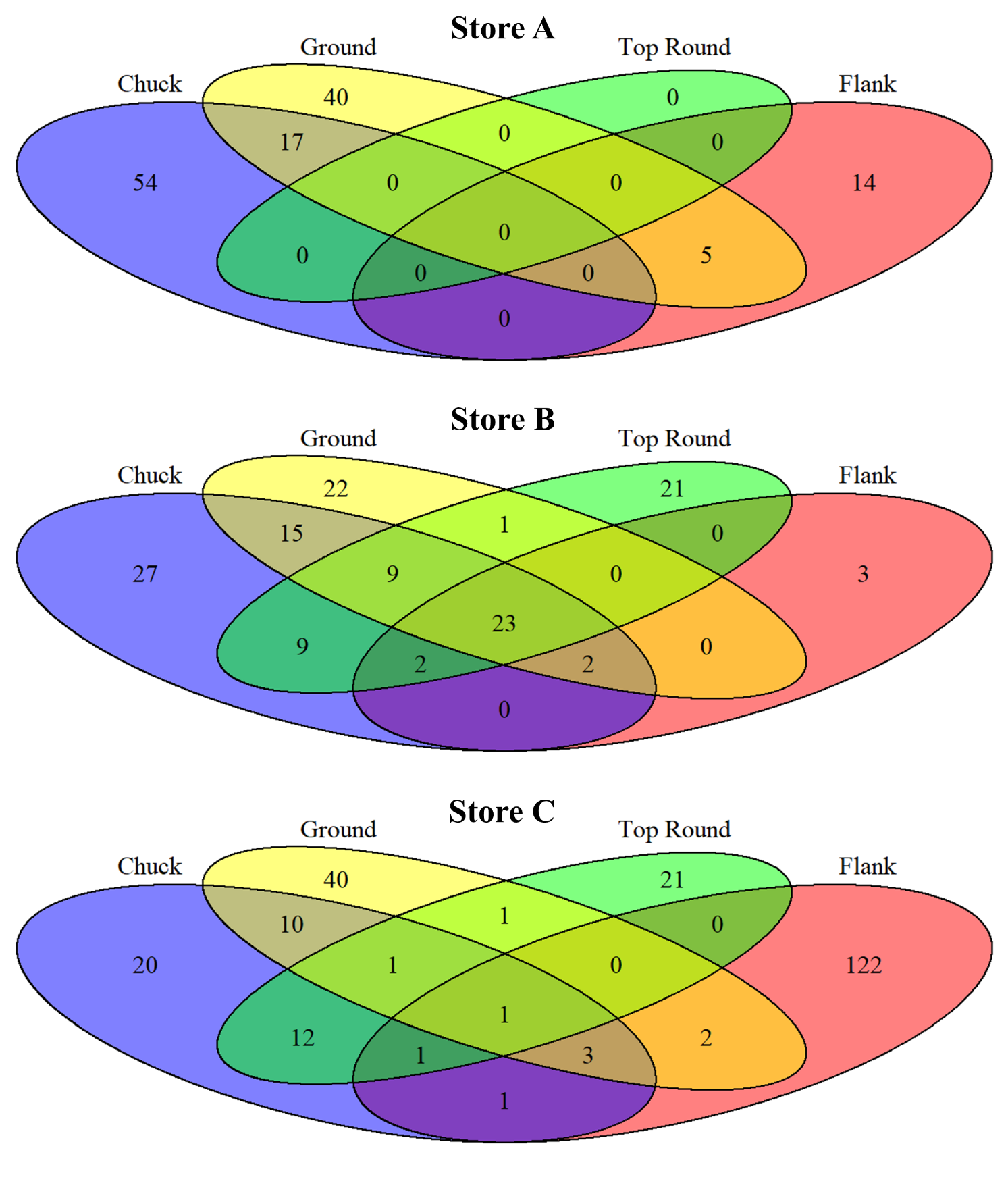


Figure S3. The number of ASVs shared among all the beef cut microbiota within each store.
